# Association between the Presence of Autoantibodies Targeting Ficolin-3 and Active Nephritis in Patients with Systemic Lupus Erythematosus

**DOI:** 10.1371/journal.pone.0160879

**Published:** 2016-09-15

**Authors:** Maëlle Plawecki, Elise Lheritier, Giovanna Clavarino, Noémie Jourde-Chiche, Saber Ouili, Stéphane Paul, Evelyne Gout, Françoise Sarrot-Reynauld, Nathalie Bardin, Pierre -Yves Boëlle, Laurent Chiche, Laurence Bouillet, Nicole M. Thielens, Jean-Yves Cesbron, Chantal Dumestre-Pérard

**Affiliations:** 1 Laboratoire d’Immunologie, pôle de Biologie, CHU Grenoble Alpes, Grenoble, France; 2 BNI team, TIMC-IMAG UMR5525 Université Grenoble Alpes, France; 3 UMR_S 1076, Vascular Research Center of Marseille, Faculté de médecine, Aix-Marseille Université, Marseille, France; 4 Centre de Néphrologie et Transplantation Rénale, CHU de la Conception, Aix-Marseille University, Marseille, France; 5 Laboratoire d’Immunologie, GIMAPEA3064, CIC1488 Vaccinology INSERM, CHU de Saint-Etienne, Saint-Etienne, France; 6 Univ Grenoble Alpes, IBS, Grenoble, France; 7 CNRS, IBS, Grenoble, France; 8 CEA, IBS, Grenoble, France; 9 Clinique Universitaire de Médecine Interne, pôle pluridisciplinaire de Médecine et de Gérontologie Clinique, CHU Grenoble Alpes, Grenoble, France; 10 Laboratoire d’Immunologie, Hôpital de la Conception, Aix-Marseille Université, Marseille, France; 11 INSERM, UMR_S 1136, Institut Pierre Louis d'Epidémiologie et de Santé Publique, Paris, France; 12 Service de Médecine Interne, Hôpital Européen, Marseille, France; Instituto Nacional de Ciencias Medicas y Nutricion Salvador Zubiran, MEXICO

## Abstract

Systemic lupus erythematosus (SLE) is a chronic autoimmune disease characterized by the production of multiple autoantibodies. Antibodies against Ficolin-3 were previously identified in the sera of some SLE patients, but their prevalence and significance have not been yet investigated. The aims of this study were to determine the prevalence of anti-ficolin-3 antibodies among SLE patients and to investigate their potential as diagnostic and/or prognostic biomarkers in SLE. In this retrospective study, sera from SLE patients (n = 165) were selected from a preexisting declared biological collection. Samples from healthy controls (n = 48) were matched with SLE sera. Disease activity was determined according to the SLEDAI score. Anti-ficolin-3, anti-dsDNA and anti-C1q antibodies levels were measured in sera by ELISA. First, a highly significant difference was found in the anti-ficolin-3 levels between SLE patients and healthy subjects. Anti-ficolin-3 antibodies were detected as positive in 56 of 165 (34%) SLE patients. The titer of anti-ficolin-3 antibodies was correlated with the SLEDAI score (r = 0.38, p<0.0001). The presence of anti-ficolin-3 antibodies was associated with anti-C1q and anti-dsDNA antibodies. Regarding associations with clinical manifestations, the presence of active lupus nephritis was significantly associated with the presence of anti-ficolin-3 antibodies (p≤0.001). This association with renal involvement was higher with anti-ficolin-3 or anti-C1q antibodies than with other auto-antibodies. Interestingly, the combination of anti-ficolin-3 and anti-C1q antibodies demonstrated higher specificity than any other serological biomarker. These results suggest that anti-ficolin-3 antibodies could be useful for the diagnosis of active nephritis in SLE patients.

## Introduction

Systemic lupus erythematosus (SLE) is a chronic autoimmune disease with a prevalence of 4/10,000 among Northern Europeans and a predisposition of women of childbearing age [1]. SLE is characterized by the presence of circulating autoantibodies directed against self-antigens, such as dsDNA, nuclear antigens and several cytoplasmic components [2]. The accumulation of the resulting immune complexes mediates a systemic inflammatory response that is the primary cause of tissue damage. Recently, defects in apoptotic cells clearance leading to secondary cell necrosis and subsequent release of intracellular autoantigens has been proposed as one of the mechanisms of induction of these autoantibodies [3] [4]. According to this proposal, molecules of importance in the uptake of dying cells could have a role in host protection against autoimmune diseases, including SLE [5] [6] [7] [8].

Apart from its well characterized anti-microbial role, the complement system has also a pivotal role in maintaining host integrity by eliminating apoptotic and damaged cells as well as by clearing circulating immune complexes [9]. Indeed, deficiencies in early complement components are often associated with increased susceptibility to both infections and autoimmune diseases such as SLE, supporting a protective role for the complement system. Mice deficient for C1q, the recognition protein of the classical complement pathway, are predisposed to SLE-like diseases [10] and human studies report that hereditary homozygous deficiencies of C1q are strongly associated with susceptibility to SLE, with a defect in apoptotic cells uptake by macrophages in SLE patients [11]. Recent reports suggest a role for mannan-binding lectin (MBL), a recognition protein of the lectin complement pathway, in the pathogenesis of SLE [12]. Indeed, gene polymorphisms leading to reduced levels of serum MBL were found associated with a predisposition to SLE [13]. However, although MBL-null mice exhibit defective clearance of apoptotic cells, they fail to develop symptoms of autoimmune diseases, suggesting the ability of other molecules to compensate for MBL deficiency [14].

The potential role of the recently identified complement-activating lectin-like proteins ficolins (ficolin-1, -2 and -3, also referred as M-, L- and H-ficolins) has been suggested, since these proteins appear to mediate immune effector functions similar to those of MBL and C1q [14] [15]. Among these proteins, Ficolin-3 deserves special attention in the context of autoimmunity since it was initially characterized as a serum antigen target (Hakata antigen) for an autoantibody present in a Japanese patient with SLE [16]. Ficolin-3 has poor lectin-like activity, as shown by glycan array screening, but it binds to acetylated albumin with high affinity [17] [18]. It has been shown to specifically recognize few bacterial polysaccharides [19] [20]. Interestingly, binding of ficolin-3 to apoptotic material has clearly been shown and has been postulated to play a role in the clearance of apoptotic debris *via* phagocytosis [21] [22]. Recently, an association of high levels of ficolin-3 with specific manifestations in SLE, but not with disease activity, was reported [23]. To date, only one study reported the presence of anti-ficolin-3 antibodies in SLE sera using a precipitation reaction for antibodies detection, and its correlation with disease activity [24].

Our study aims to investigate the presence of anti-ficolin-3 antibodies in SLE patients and to evaluate their potential as diagnostic and/or prognostic SLE biomarkers.

## Materials and Methods

### Study

This study is a multicentric non-interventional retrospective study conducted in French University Hospitals (Grenoble University Hospital and Marseille University Hospital, France) over a 3-year period (2012–2014).

### Samples

All venous blood samples were obtained from patients referred for routine detection of auto-antibodies and sera were conserved in a declared biobank DC 2012–1704 for Marseille and DC 2014–2268 for Grenoble, with respect of ethical directives.

Samples from SLE patients, satisfying the revised 1997 American College of Rheumatology (ACR) classification criteria for SLE [25] (n = 165), were selected to be further analyzed in this study. Samples from healthy blood donors (n = 48) obtained from “Etablissement Français du Sang”, matched for sex and age, served as controls. This study has been approved by local authorities (Institutional Review Board) according to standards currently applied in France (Commission Nationale de l’Informatique et des Libertés”, CNIL N°1841851vO). The study has also been registered to clinicaltrial.gov (N° NCT02625831). The study was done in accordance with the Declaration of Helsinki and Good Clinical Practice guidelines.

For SLE patients, clinical and biological manifestations as well as treatments received and disease activity evaluated using the Systemic Lupus Erythematosus Disease Activity Index (SLEDAI) [26] at the time of sampling were recorded. The SLE patients were divided into two groups for further analyses: a group with quiescent or “low disease activity” (n = 88) and a group with active or “high disease activity” (n = 77), according to the physician in charge and respectively with a SLEDAI score ≤4 and >4. All patients with renal involvement had an active lupus nephritis (LN) documented by kidney biopsy (classes I, II, III, IV or V of the ISN/RPS classification) [27]. Samples were collected just before kidney biopsy. Active proliferation was defined by the presence of at least one glomerulus showing cellular or fibro-cellular crescent or endocapillary proliferation on kidney biopsy (classes III and IV).

### Anti-ficolin-3 antibodies assessment

Recombinant ficolin-3 was purified from the culture supernatant of a stably transfected CHO cell line by affinity chromatography based on its binding capacity to acetylated BSA, as previously described [28]. Detection of anti-ficolin-3 antibodies was adapted from an ELISA previously described for measurement of anti-MBL antibodies [29]. Microtiter plates (96 wells) were coated overnight at 4°C with 2 μg/ml of recombinant ficolin-3 in 15 mM Na_2_CO_3_, 35 mM NaHCO_3_ (pH 9.6). The plates were washed three times with Phosphate Buffer Saline (PBS) containing 0.1% Tween-20 (w/v) (PBS-T) and blocked for 2 h at room temperature (RT) with PBS-T containing 1% BSA (w/v). Serum samples were added to the wells and incubated overnight at 4°C. After washing as above, horseradish peroxidase (HRP)-conjugated goat polyclonal anti-human IgGs (The Binding Site, dilution 1/15 000) were added for 1 h at RT. Subsequently, tetramethylbenzidine substrate was added to each well, the reaction was stopped by addition of H_2_SO_4_ and optical densities (ODs) were measured at 450 nm. The threshold value of 70 AU was calculated using the 98th percentile on the OD reading for the control group. A value was considered significantly elevated when the concentration was above 70 AU (arbitrary units). The concentration was defined as (OD sample/OD control) x 100 AU.

### Ficolin-3 assessment

To quantify ficolin-3 in healthy blood donors and SLE patients’ sera, an in-house ELISA was adapted from Michalski *et al*. [30]. Microtiter plates were coated with 2.5 μg/ml of mouse monoclonal anti-human ficolin-3 (mAb 334, Hycult Biotechnologies) diluted in PBS (1:400) overnight at 4°C. Plates were washed 3 times with PBS-T and blocked for 2 h with PBS-T-BSA 1% (w/v) at RT. After 3 washings with PBS-T, serum samples (1:50 dilution in PBS) or recombinant ficolin-3 were added to each well (100μl/well) at different concentrations to establish the standard curve. After 3 h incubation at 37°C, the plates were washed 4 times with PBS-T, and an in-house rabbit polyclonal anti-human ficolin-3 (1:2000 dilution in PBS-T) was added and incubated 1 h at 37°C. After 4 washings with PBS-T, HRP-conjugated rabbit polyclonal anti-ficolin-3 (1:5000 diluted in PBS-T) was added and incubated 1 h at 37°C. Detection was achieved as described above.

### Anti-dsDNA and anti-C1q antibodies assessment

Anti-double-stranded (ds)DNA antibodies were detected by ELISA (BIORAD) with normal value defined as <50 U/ml. For anti-C1q antibodies, microtiter plates (96 wells) were coated overnight at 4°C with 100 μl/well of purified C1q collagen stalks at 10 μg/ml in 20 mM H_3_BO_3_, 7.5 mM NaCl (pH 8.2) [31]. The plates were washed three times with PBS-T and blocked for 30 min at 37°C with PBS-T containing 2% BSA (w/v). Serum samples were added to the wells and incubated 30 min at 37°C. After washings as above, HRP-conjugated goat polyclonal anti-human IgG (The Binding Site, dilution 1:60,000) were added for 30 min at 37°C. Detection was achieved as described above. The threshold value was calculated using the 98th percentile on the OD reading for the control group. The concentration of IgG reactive to anti-C1q was expressed in arbitrary units (AU). The concentration was defined as (OD sample/OD cut-off) x 100 AU. A value was considered significantly elevated when the concentration was above 100 AU.

### Complement fractions assessment

Complement levels were measured by nephelometry (BNII, Siemens), according to manufacturer’s instructions, with the normal range for C4 being >100 mg/l and for C3 >880 mg/l.

### Statistics

Data analyses were carried out using Statview software version 5.0. Comparisons of continuous variables between subgroups were done using Mann-Whitney non parametric U-test. Correlations between continuous variables were assessed calculating Spearman’s rank correlation coefficients. Comparisons of categorical variables, expressed as counts (%), were done using Chi²- or Fisher’s exact-tests. P-values lower than 0.05 were considered statistically significant.

## Results

### Characteristics of SLE patients

Details on the characteristics of the 165 SLE patients and matched healthy controls are provided in Table 1. The median age of the patients was 42 years (range 16–84 years), 88% were women. At inclusion, the median SLEDAI score was 4 (range 0–24). 88 patients were classified in the “low disease activity” group and 77 in the “high disease activity” group, as previously defined (see Methods). In this latter group, 36 (47%) patients had biopsy-proven active lupus nephritis.

**Table 1:** Demographic and clinical variables for SLE patients.

Demographic variables	Control (n = 48)	SLE (n = 165)
Age, range, years	25–70	16–84
Age, mean (median) (S.D), years	41 (38) (13.3)	44 (42) (15.5)
Gender, female, n (%)	42 (88)	145 (88)
**Clinical features, n (%)**	NA	
Kidney		38 (23)
Joint		34 (21)
Haematological		10 (6)
Cutaneous		16 (10)
Cardiac		5 (3)
Pulmonary		2 (1)
Neurological		4 (2)

It should be noted that some patients may have lupus flare involving several organs. NA: not applicable.

### Prevalence of anti-ficolin-3 antibodies in SLE patients

**Fig 1:**
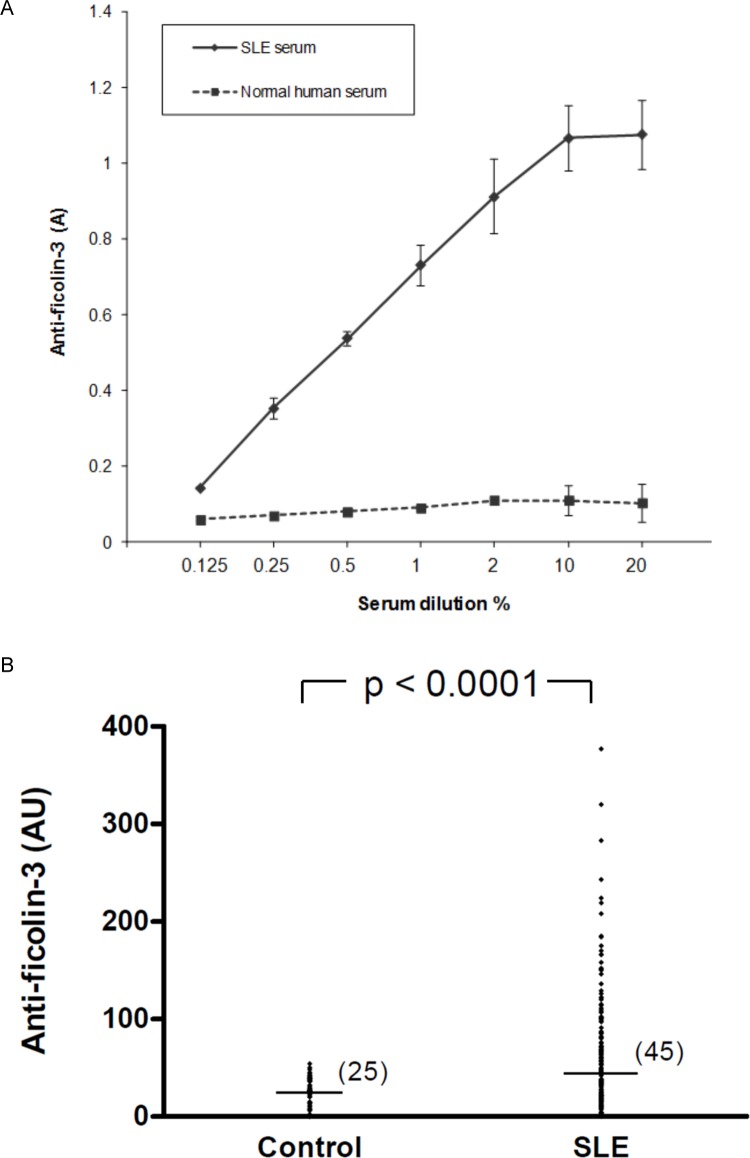
Detection of anti-ficolin-3 antibodies in patients with SLE. A) Binding of anti-ficolin-3 antibodies to immobilized ficolin-3. Microtiter plates were coated with ficolin-3. Sera from SLE patients and healthy controls were added in serial dilutions. Results represent the mean +/- standard deviation. B) Anti-ficolin-3 antibodies in serum samples. Anti-ficolin-3 antibodies were measured in 48 samples from healthy controls and in 165 samples from patients with SLE. Horizontal lines in each group indicate the median values. Statistical analyses were performed by Mann-Whitney test. A, absorbance; AU, Arbitrary units.

The presence of anti-ficolin-3 antibodies was first investigated in sera obtained from one SLE patient and one healthy blood donor. A dose-dependent binding of IgG to solid-phase ficolin-3 was demonstrated, while serum from a healthy blood donor did not show significant reactivity (Fig 1A). Those sera were used as positive and negative standards for further experiments. As a control, ELISA wells were coated with BSA and no detectable binding was observed for both sera.

Subsequently, anti-ficolin-3 antibodies were tested in serum samples from the 165 SLE patients and from 48 healthy controls. Titers of anti-ficolin-3 antibodies were significantly higher in SLE patients than in healthy controls (median 45 vs 25 AU, p<0.0001, Mann-Whitney U-test) (Fig 1B). Using a cutoff value of 70 AU (see Methods) anti-ficolin-3 antibodies were detected as positive in 56 (34%) of 165 SLE patients. No anti-ficolin-3 antibodies were found in patients with rheumatoid arthritis (n = 14), Sjögren’s syndrome (n = 12), chronic renal failure (n = 12) and IgA nephropathy such as Berger disease (n = 12) (S1 Fig).

### Association between anti-ficolin-3 antibodies levels and clinical and biological SLE activity

**Fig 2:**
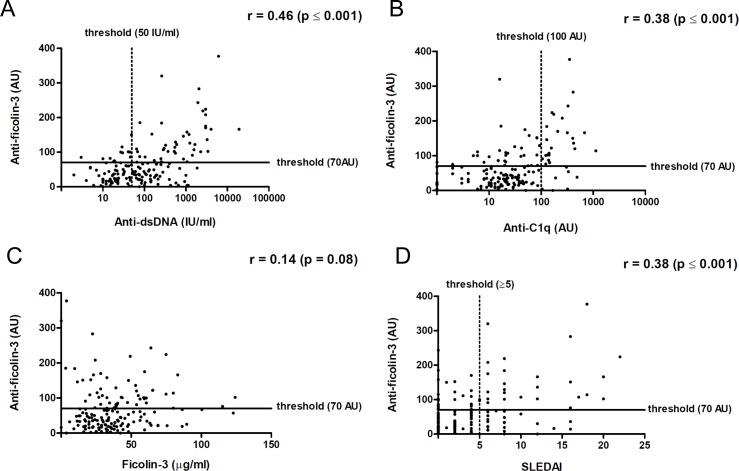
Association between anti-ficolin-3 antibodies titers, biological markers and disease activity in patients with SLE. Association between anti-ficolin-3 titers and anti-dsDNA antibodies (A), anti-C1q antibodies (B) and ficolin-3 concentrations (C) in SLE. D) Association between anti-ficolin-3 titers and SLE Disease Activity Index (SLEDAI). Statistical analyses were performed by Spearman’s rank correlation test.

In SLE patients, the titers of anti-ficolin-3 antibodies were significantly associated with the titers of anti-dsDNA antibodies (r = 0.46, p<0.0001, Spearman test) (Fig 2A) as well as with the titers of anti-C1q antibodies (r = 0.38, p<0.0001, Spearman test) (Fig 2B). The presence of anti-ficolin-3 antibodies was not correlated to ficolin-3 serum levels (r = 0.14, p = 0.08, Spearman test) (Fig 2C). Furthermore, a statistical difference in serum ficolin-3 concentrations was observed between SLE patients and healthy individuals (p = 0.015, Mann-Whitney U-test) (S2 Fig).

A significant association between anti-ficolin-3 antibodies titers and disease activity (SLEDAI) was observed (r = 0.38, p<0.0001, Spearman test) (Fig 2D). Similar results were obtained using a clinical SLEDAI, which does not take anti-dsDNA antibodies and complement titers into account. A significant association was also observed between SLEDAI score and titers of anti-dsDNA antibodies (r = 0.62, p<0.0001, Spearman test) or titers of anti-C1q antibodies (r = 0.38, p<0.0001, Spearman test). Of note, ficolin-3 levels were not associated with lupus activity evaluated by SLEDAI score (r = 0.296, p = 0.083, Spearman test) (S3 Fig).

### Association between SLE-related clinical features and autoantibodies positivity

Among various clinical manifestations presented by our patients (Table 2), the presence of anti-ficolin-3 antibodies was significantly associated with lupus nephritis (p≤0.001, Chi2 test). Similarly, the presence of anti-C1q antibodies was also significantly associated with lupus nephritis (p≤0.001, Chi2 test). Anti-dsDNA antibodies and low complement C3 and/or C4 were less associated with kidney involvement (respectively p = 0.012 and p = 0.015, Chi2 test).

**Table 2:** Associations between SLE-related clinical features of 77 patients with active SLE and titers of anti-ficolin-3, anti-C1q and anti-dsDNA antibodies.

SLE damages	Number of patients with flare	Anti-ficolin-3		Anti-C1q		Anti-dsDNA		Low complement	
		Chi-2	p	Chi-2	p	Chi-2	p	Chi-2	p
Kidney	36	10.92	*** ≤ 0.001	12.72	*** ≤ 0.001	6.36	* 0.012	5.87	* 0.015
Joint	33	3.90	* 0.048	10.90	*** ≤ 0.001	5.53	* 0.019	10.84	*** ≤ 0.001
Haematological	8	0.002	0.970	1.62	0.203	0.10	0.756	2.60	0.107
Cutaneous	16	7.57	** 0.006	1.73	0.188	6.47	* 0.011	0.03	0.861
Cardiac	5	0.19	0.665	0.14	0.710	0.002	0.965	0.14	0.709
Pulmonary	2	2.11	0.147	4.27	* 0.039	0.54	0.463	0.003	0.955
Neurological	4	0.001	0.979	0.11	0.743	2.19	0.139	1.23	0.268

It should be noted that some patients may have lupus flare involving several organs.

*P≤0.05

**P≤0.01

***P≤0.001 (Chi2-test).

### Anti-ficolin-3 antibodies and active lupus nephritis

**Fig 3:**
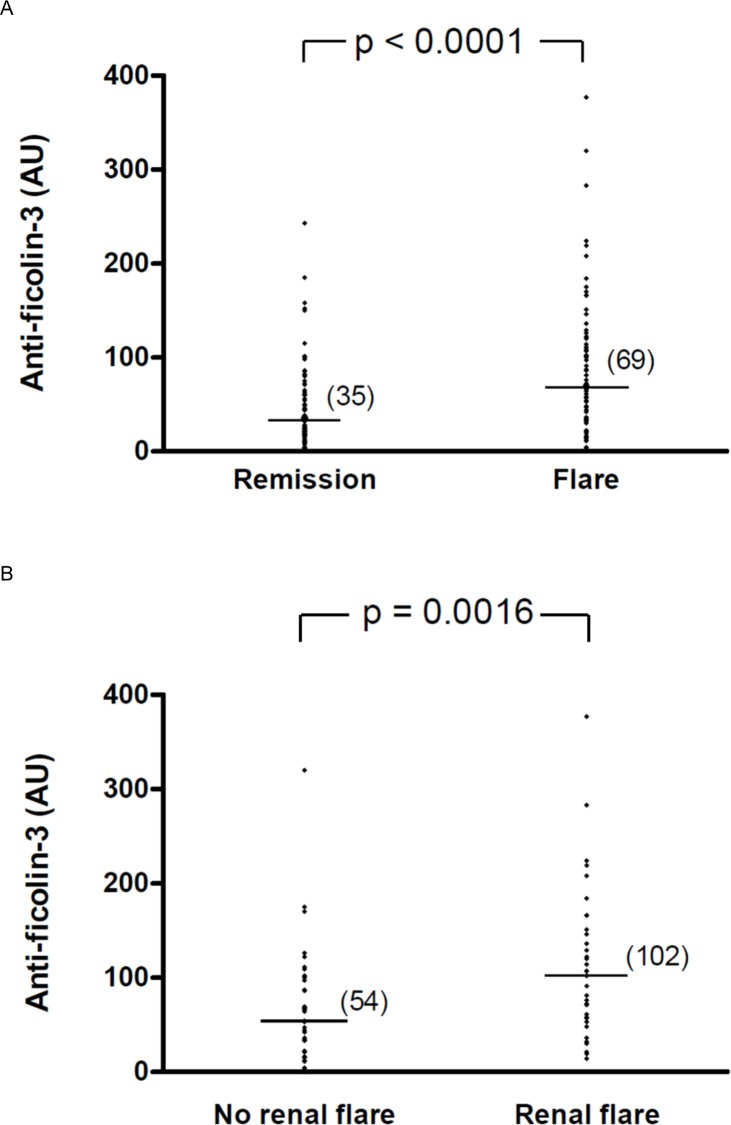
Serum anti-ficolin-3 antibodies titers in SLE patients with active disease (flare) or in disease remission. A) Anti-ficolin-3 titers in SLE patients with active disease (SLEDAI >4) (n = 77) or in disease remission (SLEDAI ≤4) (n = 88). B) Anti-ficolin-3 titers in SLE patients with active disease with renal involvement (n = 36) or without renal involvement (n = 41). Horizontal lines in each group indicate the median values. Statistical analyses were performed by Mann-Whitney test.

Anti-ficolin-3 antibodies were detected as positive in 38 (49%) of the 77 SLE patients characterized as having “high disease activity”. Titers of anti-ficolin-3 antibodies were significantly higher in SLE patients with “high disease activity” than in those with “low disease activity” (median 69 vs 35 AU, p<0.0001, Mann-Whitney U-test), (Fig 3A). Anti-ficolin-3 antibodies were detected as positive in 25 (69%) of 36 SLE patients with active disease and lupus nephritis. Characteristics of the patients are shown in Table 3. Interestingly, titers of anti-ficolin-3 antibodies were significantly higher in this subset of SLE patients with active disease and lupus nephritis than in patients with active disease but without renal involvement (median 102 vs 54 AU, p = 0.0016, Mann-Whitney U-test) (Fig 3B).

**Table 3:** Characteristics of SLE patients with lupus nephritis.

**Demographic data**	Age, mean +/- SD	36 +/- 14.6
	Male/Female, n (%)	12 (33) / 24 (67)
	White/African/Asian, n (%)	30 (83)/5 (14)/1 (3)
**Renal abnormalities**	Urinary casts (heme-granular or RBC casts)	24 (69)
n (%)	High blood pressure	6 (17)
	Proteinuria	34 (94)
	Abnormal creatinine	13 (36)
**ISN/RPS 2003 class**	I	2 (6)
n (%)	II	1 (3)
	III	4 (11)
	IV	23 (64)
	V	6 (17)
**Therapy**	Corticoids	21 (58)
n (%)	Azathioprine	3 (8)
	Plaquenil	13 (36)
	Methotrexate	1 (3)
	Rituximab	2 (6)
	Mycophenolate mofetil	3 (8)

RBC, red blood cells

### Lupus nephritis and combined biomarkers

As anti-ficolin-3, anti-C1q, anti-dsDNA and low complement C3 and/or C4 were associated with renal disease activity, we characterized the diagnostic performances of these biomarkers, alone or in combination, in our cohort. Sensitivity and specificity of anti-ficolin-3 antibodies for SLE renal involvement were respectively 70% and 68%. Predictive positive and negative values were respectively 66% and 72%. While anti-dsDNA antibodies alone indicated a better sensitivity than the other biomarkers, interestingly the combination of anti-ficolin-3 and anti-C1q antibodies showed the higher specificity for renal involvement (Table 4).

**Table 4:** Performances of SLE biomarkers.

Biomarkers	Renal flare	No renal flare	Sensitivity (%)	Specificity (%)	NPV (%)	PPV (%)
Anti-ficolin-3 Abs	25/36 (70%)	13/41 (32%)	70	68	72	66
Anti-dsDNA Abs	33/36 (92%)	28/41 (68%)	92	32	81	54
Anti-C1q Abs	19/36 (53%)	6/41 (15%)	53	85	67	76
Low complement	24/36 (67%)	16/41 (39%)	66	61	68	60
Anti-ficolin-3 and anti-C1q Abs	18/36 (50%)	3/41 (7%)	50	93	68	86
Anti-ficolin-3 and anti-C1q Abs and low complement	15/36 (42%)	3/41 (7%)	42	93	64	83

Abs, antibodies; NPV, negative predictive value; PPV, positive predictive value.

We then analyzed the possible link between the presence of anti-ficolin-3 antibodies and immunohistological characteristics of the 36 kidney biopsies. Patients with active proliferative lupus nephritis (i.e. Classes III and IV) had significantly more positive anti-ficolin-3 and anti-C1q antibodies than those with non-proliferative lupus nephritis (respectively p = 0.001 and p = 0.006, Fisher’s exact test) (Table 5). Other biological data such as proteinuria, abnormal creatinine, low complement and anti-DNA antibodies were not or little significantly different between the two groups.

**Table 5:** Biological characteristics of patients with active and inactive lupus nephritis.

	LN with non active proliferation n = 9 (%)	LN with active proliferation n = 27 (%)	*P*
Proteinuria	9 (100)	25 (93)	1.00
Abnormal creatinine	1 (11)	12 (44)	0.11
Low complement	3 (33)	21 (78)	0.04 *
Anti-dsDNA Abs	7 (78)	26 (96)	0.15
Anti-C1q Abs	1 (11)	18 (67)	0.006 **
Anti-ficolin-3 Abs	2 (22)	23 (85)	0.001 ***

*P≤0.05

**P≤0.01

***P≤0.001 (Fisher’s exact-test)

## Discussion

Our study is to our knowledge the first one assessing the presence of antibodies targeting ficolin-3 and measuring their titers using an ELISA method in SLE patients. Indeed, only one previous study reported the presence of auto-antibodies against ficolin-3 in lupus sera, but authors used a double immunodiffusion technique which was not quantitative [24]. In that study, only 4 among 110 SLE patients (3.6%) were found to have anti-ficolin-3 antibodies. Conversely, in our cohort, 57/165 (34%) patients were found positive. This discrepancy likely relies on the higher sensitivity of our ELISA over older techniques.

The present study is also the first to investigate association between anti-ficolin-3 antibodies and clinical and serologic parameters of SLE using a large cohort of SLE patients. First, we found that the anti-ficolin-3 autoantibodies levels were significantly higher in SLE patients as compared to healthy subjects. Interestingly, these antibodies were found negative in other autoimmune diseases such as rheumatoid arthritis and Sjögren’s syndrome. Then we assessed the presence of anti-ficolin-3 antibodies in sera of SLE patients during inactive and active phases of the disease. The levels of anti-ficolin-3 were significantly higher in patients with active than quiescent disease and we observed a statistical correlation of antibodies levels and the global activity of the disease evaluated by the SLEDAI score.

These findings are of importance. Indeed, while many tests exist for the detection of autoantibodies in the sera of patients with SLE, such as anti-dsDNA and anti-C1q [32], only few of them are used in clinical practice. Most of the studies indicated that anti-dsDNA antibodies could be useful markers of the overall activity of SLE [33]. Some considered the elevated levels of anti-dsDNA as indicative of active forms of SLE [34]. In accordance with those observations, titers of anti-dsDNA were correlated with lupus activity in our SLE cohort. However, some studies suggested that anti-dsDNA antibodies have to be used with caution to evaluate the SLE activity [35] [36]. Because the associations between antibody profile and clinical symptoms are unclear, it is crucial to find new relevant diagnostic and/or prognostic biomarkers.

Similarities in structure and function exist between collagen-like defense molecules such as MBL, C1q and ficolin-3. Anti-MBL antibodies were found in SLE patients’ sera but no significant relationship was observed with clinical characteristics of SLE [29] [37]. Several studies reported that anti-C1q levels did not correlate with SLEDAI score in SLE patients [36] [38]. However, in our study, we found an association between titers of anti-C1q and SLE disease activity, in accordance with the study of Moura *et al*. [39]. The occurrence of anti-C1q antibodies in patients with active lupus nephritis remains controversial but the absence of anti-C1q antibodies seems to exclude active renal disease [40] [41].

Lupus nephritis, a severe manifestation of SLE, affects up to 30 to 50% patients in the course of the disease. Interestingly, in our cohort we observed a very high prevalence (70%) of anti-ficolin-3 antibodies in the subset of SLE patients with lupus nephritis. Regarding clinical associations, anti-ficolin-3 antibodies positivity was significantly related to renal involvement. There was no significant association between the positivity of anti-ficolin-3 antibodies and other clinical characteristics of SLE such as joint, hematological, neurological, cardiac or pulmonary damages. This is in accordance with our results showing significantly higher anti-ficolin-3 titers in patients with active nephritis compared to those in patients with active disease but without renal involvement. Moreover, the possibility that renal dysfunction might lead to higher level of antibody has been eliminated since anti-ficolin-3 antibodies were found negative in dialyzed patients with chronic renal failure. Importantly, we found that anti-ficolin-3 antibodies were more closely associated with active lupus nephritis than any other auto-antibodies. The development of nephritis is a major determinant of disease morbidity and mortality. Thus, the identification of non-invasive biomarkers in lupus nephritis is a priority. We observed that the combination of anti-ficolin-3 and anti-C1q antibodies outperformed anti-C1q, anti-dsDNA or low complement alone, demonstrating a higher specificity than either serological biomarker. Until now, anti-C1q in combination with anti-dsDNA and low complement was reported as the strongest serological association with renal involvement. Orbai et *al*. and Yang et *al*. showed that the combination of anti-C1q and anti-dsDNA was associated with higher renal disease activity [42] [43]. Even if nephritis assessment based on standard parameters such as proteinuria and creatinine remains essential, the combination of anti-ficolin-3 and anti-C1q antibodies could open new perspectives in evaluation of lupus nephritis.

Although a causal relationship between the presence of anti-ficolin-3 antibodies and lupus nephritis remains to be determined, the possible role of anti-ficolin-3 antibodies in the pathogenesis of lupus is a key issue. On one hand, anti-ficolin-3 antibodies may contribute to the formation of circulating immune complexes that are deposited in the kidney or to the local formation of immune complexes on the glomerular basement membrane. On the other hand, anti-ficolin-3 antibodies from SLE patients could specifically target ficolin-3 bound to apoptotic cells, thereby altering the clearance of dead cells, which is an important hypothesis of the pathogenesis of SLE. This role of anti-ficolin-3 antibodies is plausible, because ficolin-3 seems not to be affected in SLE. Indeed, we found increased serum levels of ficolin-3 in patients with SLE and no association of ficolin-3 levels and lupus activity, as shown by Andersen *et al*. and Hein *et al*. [44] [23]. Therefore, the role of anti-ficolin-3 antibodies in the SLE pathogenesis remains to be investigated and an interesting point resides in identifying the auto-antibodies specificities against ficolin-3. Indeed, anti-ficolin-3 antibodies could recognize either the collagen-like or the fibrinogen-like domains of ficolin-3 with different outcomes. A significant participation of complement activation was reported in the pathogenesis of lupus nephritis. The alternative and lectin pathways were involved in the progression of glomerular injury of patients with lupus nephritis [45] and in situ deposition of complement components in renal biopsy specimens has been shown [46]. Ficolin-3, which is a strong activator of the lectin pathway, could also contribute to renal pathogenesis. Moreover, Pang *et al*. [47] showed that in the presence of anti-C1q antibodies, immune complexes would not be removed from the circulation and might prone to deposit in some organs, such as kidneys, and induce inflammatory injury. The potential role of anti-ficolin-3 antibodies was not discussed in the literature and it would be of interest to study the association between anti-ficolin-3 antibodies and specific pathological lesions on kidney biopsies. In this regard, a link between the presence of anti-ficolin-3 antibodies and the immunohistological characteristics of the kidney biopsies was observed. However further investigations would be necessary to confirm these findings in a larger cohort of patients with lupus nephritis.

In conclusion, this is the first report on the significant presence of anti-ficolin-3 antibodies in the serum of SLE patients. From a clinical point of view, these data support the usefulness of anti-ficolin-3 as an additional serological biomarker for the diagnosis of active lupus with renal manifestation, but additional longitudinal studies are needed to validate the diagnostic and/or prognostic role of this new SLE parameter.

## Supporting Information

S1 Fig Detection of anti-ficolin-3 antibodies in patients with Sjögren’s Syndrome and Rheumatoid Arthritis. Microtiter plates were coated with ficolin-3. Sera from healthy controls (n = 48), Sjögren’s Syndrome (SS) (n = 12), Rheumatoid Arthritis (RA) (n = 14), chronic renal failure (n = 12), IgA nephropathy such as Berger disease (n = 12) and SLE patients were added in serial dilutions. Horizontal lines in each group indicate the median values. AU, arbitrary units.
(TIF)

S2 Fig Ficolin-3 levels in serum samples. Ficolin-3 was measured in 48 samples from healthy controls and in 165 samples from patients with SLE. Horizontal lines in each group indicate the median values. Statistical analyses were performed by Mann-Whitney test.
(TIF)

S3 Fig Association between ficolin-3 levels and Disease Activity Index (SLEDAI) in patients with SLE. Statistical analyses were performed by Spearman’s rank correlation test.
(TIF)
