# Supplementary figures and images for: Association between the Presence of Autoantibodies Targeting Ficolin-3 and Active Nephritis in Patients with Systemic Lupus Erythematosus
Source: PLoS One. 2016 Sep 15;11(9):e0160879. doi: 10.1371/journal.pone.0160879 (PMC5025237; doi:10.1371/journal.pone.0160879)

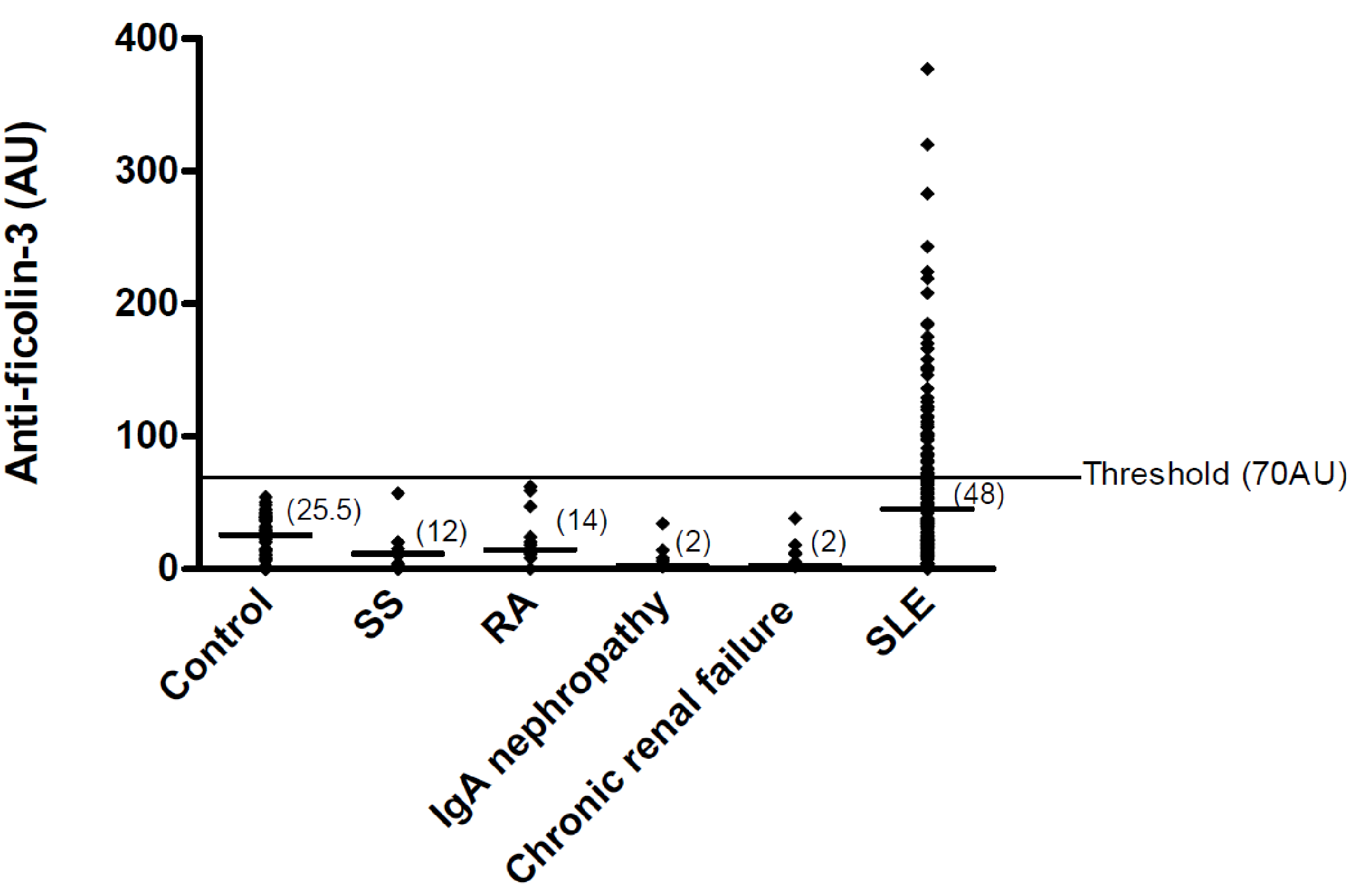

Supplement: S1 Fig — Microtiter plates were coated with ficolin-3. Sera from healthy controls (n = 48), Sjögren’s Syndrome (SS) (n = 12), Rheumatoid Arthritis (RA) (n = 14), chronic renal failure (n = 12), IgA nephropathy such as Berger disease (n = 12) and SLE patients were added in serial dilutions. Horizontal lines in each group indicate the median values. AU, arbitrary units. (TIF) [file pone.0160879.s001.tif]

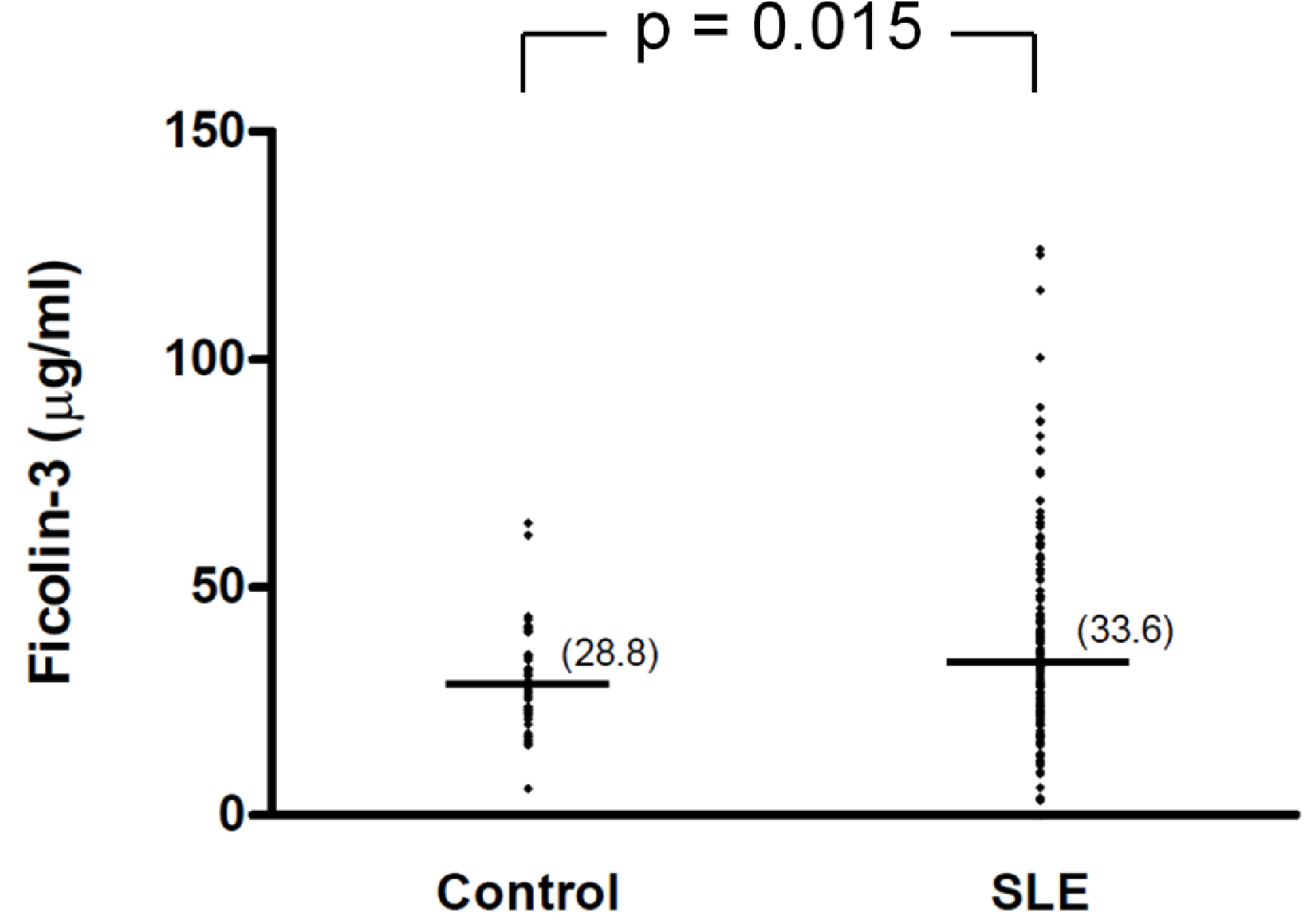

Supplement: S2 Fig — Ficolin-3 was measured in 48 samples from healthy controls and in 165 samples from patients with SLE. Horizontal lines in each group indicate the median values. Statistical analyses were performed by Mann-Whitney test. (TIF) [file pone.0160879.s002.tif]

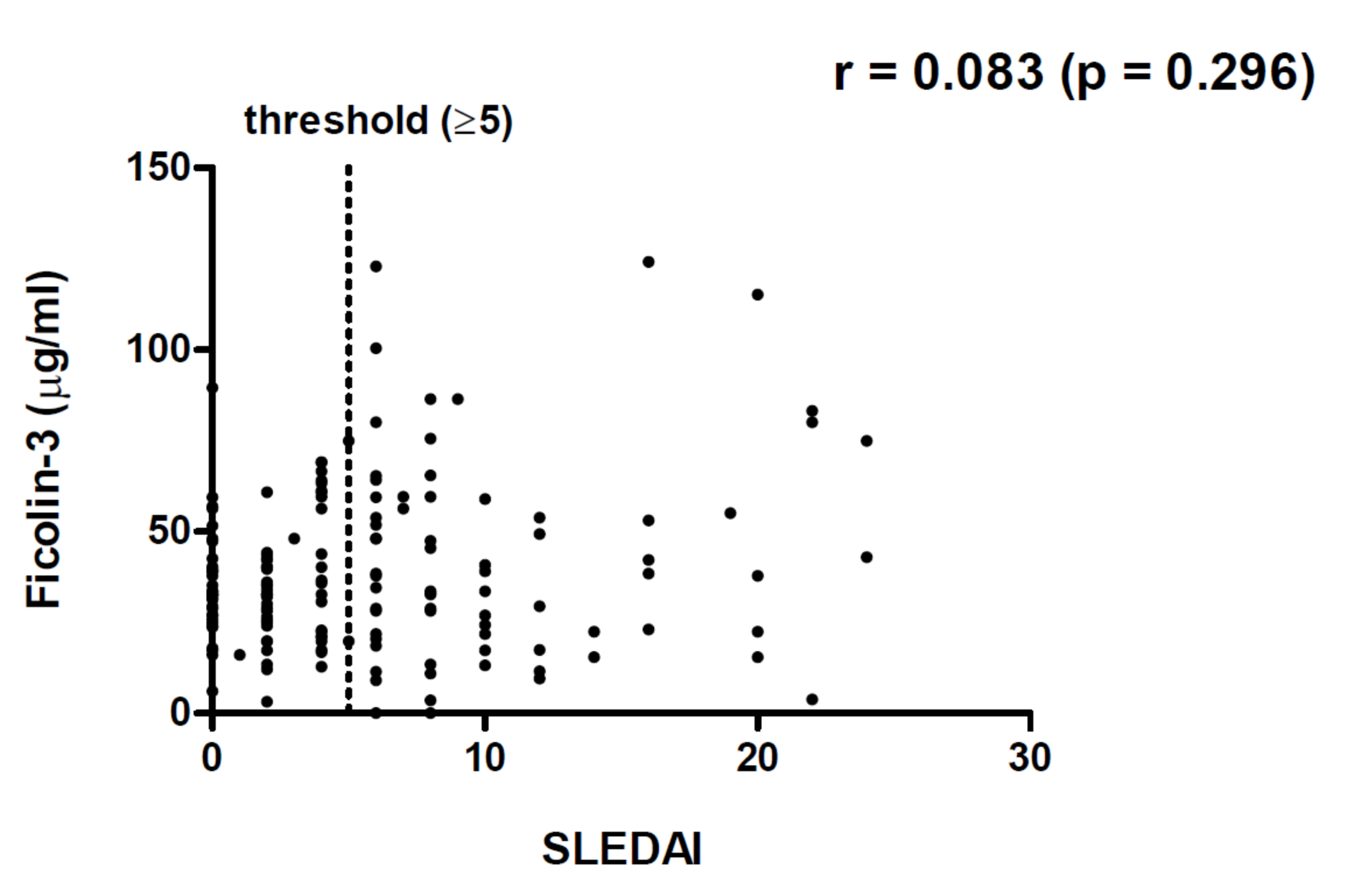

Supplement: S3 Fig — Statistical analyses were performed by Spearman’s rank correlation test. (TIF) [file pone.0160879.s003.tif]
